# Supplementary material for: Danish dog owners’ use and the perceived effect of unlicensed cannabis products in dogs
Source: PLoS One. 2024 Jan 31;19(1):e0296698. doi: 10.1371/journal.pone.0296698 (PMC10830036; doi:10.1371/journal.pone.0296698)
Supplement: S1 Appendix — The questionnaire has for this publication been translated from the original survey language (Danish). (PDF) [file pone.0296698.s001.pdf]

S1 Appendix. Survey questionnaire.

The use of cannabinoids for canine medical conditions among Danish dog owners

Thank you for participating in this online survey from the University Hospital for Companion Animals, University of Copenhagen.

The questionnaire takes 2-3 minutes to complete and consists of up to 8 questions.

As a possible starting point for future research in veterinary cannabis usages and effect in dogs, we have initiated this survey. The aim is to uncover the use of cannabis in Danish dogs.
The survey is completely anonymously and it will therefore not be possible to identify you or your dog through the answers provided.

By completing this questionnaire, you accept that we can use this anonymized data for future research and publication.

Which region are you located in?

(1) ❑ Greater Copenhagen
(2) ❑ Northern Zealand
(3) ❑ Other Zealand
(4) ❑ Funen
(5) ❑ Northern Jutland
(8) ❑ Mid Jutland
(6) ❑ Southern Jutland
(7) ❑ Islands

What size breed is your dog?

(1) ❑ Small breed <10 kg
(2) ❑ Medium breed 10-20 kg
(3) ❑ Large breed 20-30 kg
(4) ❑ Extra-large breed or Giant breed > 30 kg

Have you treated your dog with one of the following?

(1) ❑ CBD drops/oil
(2) ❑ CBD capsules
(3) ❑ CBD ointment/cream
(4) ❑ CBD powder
(5) ❑ CBD spray
(6) ❑ Products primarily containing THC
(7) ❑ Cannabis tea
(8) ❑ Other formulations of cannabis/hemp products, please specify_____
(9) ❑ No, none of the above

What was the intent of the treatment?

(1) ❑ Treatment of cancer
(2) ❑ Treatment of pain
(3) ❑ Treatment of poor appetite
(4) ❑ Treatment of gastrointestinal disease or symptoms
(5) ❑ Disease prevention or for well-being purposes
(6) ❑ Treatment of allergy
(7) ❑ Treatment of seizures
(8) ❑ Alternative purposes, please specify_____

Was there an effect of the treatment?

(1) ❑ Yes, very convincing and good effect
(2) ❑ Yes, convincing with some effect
(3) ❑ Possible, but not convincing effect
(4) ❑ No, no effect

Where did you purchase the product?

(1) ❑ Internet
(2) ❑ Pharmacy
(3) ❑ Herbalist
(4) ❑ Abroad
(5) ❑ At a marked
(6) ❑ Other place, please specify_____

Have your dog received treatment with other herbal/natural remedies?

(1) ❑ Have used or uses herbal remedies
(2) ❑ Have not used herbal remedies

Describe what herbal remedies you have supplemented your dog with

(1) ❑ Tick of the box, and please specify_____
