# Supplementary material for: Danish dog owners’ use and the perceived effect of unlicensed cannabis products in dogs
Source: PLoS One. 2024 Jan 31;19(1):e0296698. doi: 10.1371/journal.pone.0296698 (PMC10830036; doi:10.1371/journal.pone.0296698)
Supplement: S2 Appendix — The list has for this publication been translated from the original survey language (Danish). (PDF) [file pone.0296698.s002.pdf]

S2 Appendix. Overview of reported use of herbal remedies by respondents. The list has for this publication been translated from the original survey language (Danish).

Aloe Vera
Alpha-casozepine
Amino acids (L-Tryptophan, L-Theanine)
Bach Flower Remedies
B-vitamin
C-vitamin
Chinese herbs
Chlorophyll tablets
Chondroitin
Collagen
Colloidal Silver
Cranberry extract
D-mannose
Essential oils (Omega 3/6)
Fish oil
Garlic powder
Ginger
Glucosamine
Gold
Green-lipped Mussels
Homeopathy
Honey wound dressings
Hyaluronic acid
Milk Thistle
Pheromones
Prebiotics
Probiotics
Psyllium
Rosehip powder
See weed powder
