# Supplementary material for: Danish dog owners’ use and the perceived effect of unlicensed cannabis products in dogs
Source: PLoS One. 2024 Jan 31;19(1):e0296698. doi: 10.1371/journal.pone.0296698 (PMC10830036; doi:10.1371/journal.pone.0296698)
Supplement: Attachment — Submitted filename: Response to Reviewers.pdf [file pone.0296698.s004.pdf]

3RD OF MARCH 2023 AND 29TH OF FEBRUARY 2021

Dear Editors

The authors would like to thank the reviewers and section editor for their careful reading and thoughtful comments that helped improve the quality and clarity of the manuscript. We thank the reviewers for the time and effort taken to review our study, and for their insightful and encouraging comments.

We have addressed all the comments and have edited the manuscript to hopefully satisfy the raised concerns.

Response to Editor

1. *Please ensure that your manuscript meets PLOS ONE's style requirements, including those for file naming.*
   The style requirements have been reassessed and corrected.

2. *In your Methods section, please provide additional information about the participant recruitment method and the demographic details of your participants. Please ensure you have provided sufficient details to replicate the analyses such as:*

   a) the recruitment date range (month and year)
   We have changed the original wording ("The final questionnaire was distributed as an online survey from the 5th of July 2019 to the 27th of November 2019.") to "The final questionnaire was distributed as an online survey from July 2019 to November 2019."

   b) a description of any inclusion/exclusion criteria that were applied to participant recruitment,

The inclusion and exclusion criteria have been emphasized by adding the following; "The inclusion criteria for participation were dog owners located in Denmark. The registrations from respondents not completing the questionnaire were excluded from the analysis."

c) a table of relevant demographic details,
A demographic table has been added to the manuscript.

d) a statement as to whether your sample can be considered representative of a larger population,
The sample is a self-selected convenience sample with a high probability of bias due to the subject. We do expect that respondents in this survey either are highly pro or con towards the subject, which could reflect motivation for participation. Therefore, the sample population is not considered representative of the general population.

e) a description of how participants were recruited, and
Kindly make attention to lines 103-105: "The respondents were a self-selected convenience sample who accessed the survey via a link posted on the University of Copenhagen's Hospital for Companion Animals' Facebook page, as well as via distribution to other Danish Facebook groups for dog owners".

f) descriptions of where participants were recruited and where the research took place.
We have revised the paragraph with detailed recruitment and location specifications; "The survey was designed and reviewed by the co-investigators, and an online pilot questionnaire was tested by staff at the University of Copenhagen (UCPH). They provided feedback in an iterative process and the pilot questionnaire was adjusted for inappropriate branching and flow, as well as misleading reply options. The study originated from UCPH…".

3. *Has the statistical analysis been performed appropriately and rigorously?*
   Reviewer #1: No
   Reviewer #2: No
   The statistics were discussed with our statistical advisors and have been revised.

4. *Have the authors made all data underlying the findings in their manuscript fully available?* *The PLOS Data policy requires authors to make all data underlying the findings described in their manuscript fully available without restriction, with rare exception (please refer to the Data Availability Statement in the manuscript PDF file). The data should be provided as part of the manuscript or its supporting information, or deposited to a public repository. For example, in addition to summary statistics, the data points behind means, medians and variance measures should be available. If there are restrictions on publicly sharing data—e.g. participant privacy or use of data from a third party—those must be specified.*

Reviewer #1: No

Reviewer #2: No
We do apologize for this error. The complete dataset has been supplied in the original language and as an English translation as supportive information.

Response to Reviewer #1

An interesting and important contribution to a rapidly changing field of research. So long as the ethics approval process is considered sufficient for this journal, I suggest a major revision is needed. The paper needs to be streamlined, data presentation and analysis need to be revised, and the survey's findings should be better situated within the literature.
Thank you for the very detailed, insightful comments and recommendations. We are very appreciative of the extensive considerations and valuable comments. To the best of our ability, we have revised the manuscript.

I am unsure whether the ethics approval process for this study is considered adequate for this journal. There was no explicit consent form for the survey; consent was obtained tacitly by a clause in the introduction to the anonymous survey stating that, "By completing this questionnaire, you accept that we can use this anonymized data for future research and publication".
Thank you for the very relevant concern. At the University of Copenhagen, a Data Protection Officer advises employees on the rules of data protection in regards to scientific data collection. The Officer did not find the survey including any personal data or sensitive data.
At the University of Copenhagen, a local Care and Use Committee (The Local Ethical and Administrative Committee) reviews and approves all studies performed at the Department of Veterinary Sciences. They have on application, prior to the study was performed, concluded that we did not need ethical approval, due to the nature of the data and respondent anonymity, and categorized the study as exempt.
After your relevant comments, we have gone back to the Local Ethical and Administrative Committee and asked them to comment on this. They have revised their conclusion and have approved the study (study identification number: 2020-20).

The authors have stated that they have made the data available, but the raw data are not available; only summary statistics are currently available in the manuscript and supplementary files.
We do apologize for this error. The original and translated data have been included in the revised edition.

Overall, I think this paper has the potential to make a solid contribution to the current literature. In my view, the paper should remain more tightly focused on the following two survey findings:
1) owners have administered cannabinoids to their dogs for a wide range of medical conditions (regardless of legal status).
2) owners perceive a range of efficacy when treating their dogs for these indications (which could

be confounded by inappropriate dosing, content or ratio of THC:CBD, placebo effect, ineffective products, or inappropriate formulation for disease condition).

Thank you for your encouraging comments. The manuscript has been revised to meet your suggestions.

The emphasis on the following two peripheral survey findings is problematic:
3) owners used different formulations of cannabis-based products.
4) owners perceived a greater efficacy of cannabis-based products in smaller breed sizes.
The two paragraphs that follow here explain, among other things, why the discussion of these two survey findings needs to be substantially reworked or in some cases removed altogether, with far less emphasis placed on them than in the current version of the manuscript.

As an exploratory study based on an anonymous online survey, it is most appropriate to restrict statistical analyses (if you use them at all) to one or two important hypotheses, rather than trying to assess the different relationships between a number of independent variables and the outcome of interest (i.e., owner-perceived efficacy). Without adequate information on relevant confounders (e.g., exact formulation, correct dose or not), it is not possible to simultaneously assess the impact of all of these independent variables together, while controlling for possible confounding effects (as you would with multivariable ordinal logistic regression). Therefore, in addition to the analysis of cannabinoid use in dogs by geographic region, my suggestion would be to restrict the use of statistical analyses to assessing the potential association between owner-perceived efficacy and the treatment indication, making sure to emphasize the limitations of this analysis in the discussion (i.e., there are other possible explanations for why the owner did not perceive an effect).

Thank you very much for this very relevant suggestion. We have revised and changed all statistical analyses.

In addition, I would suggest cross-tabulating the type of formulation by indication for the indication groups that you analyze statistically, and providing the descriptive statistics for this in text. For example, what is the distribution of formulation types for the treatment of pain? It's relevant to include this, since the formulation represents a potential confounder for the owner's perceived efficacy.

Thank you for this suggestion. We find that there are so few respondents using other formulations than CBD oil that we are unsure if providing these data separately is informative and therefore have not included it in the manuscript. The cross-tabulation is inserted beneath to back-up our point of view.

	CBD oil		CBD Capsules		CBD Creme		CBD Powder		Spray		THC		Tea		Other		Total
	n	%	n	%	n	%	n	%	n	%	n	%	n	%	n	%	
Pain	231	88	9	3	3	1	3	1	2	1	6	2	0	0	8	3	262
Behavioral issue	93	94	0	0	0	0	0	0	1	1	0	0	0	0	5	5	99
Allergy	43	84	0	0	5	10	0	0	0	0	1	2	0	0	2	4	51

The analyses with "breed size" and "formulation type" are arguably not particularly meaningful as they are more prone to confounding bias and ambiguity. Most relevant here is the fact that in the absence of dosing data, any conclusions drawn concerning a relationship between owner-perceived efficacy and either breed size or formulation type has the potential to be seriously confounded.

Moreover, although the analysis with "breed size" revealed a trend, I am concerned about a potential ambiguity in the survey question here, since both "breed size" and weight (in kg) are listed as possible responses: in a large-breed puppy, the dog's eventual weight, or their "breed size", in this sense, may have been recorded by respondents, as opposed to simply the dog's current weight, irrespective of breed.

Thank you for pointing out this very obvious concern. The original question in Danish does not include the word “breed”, but just “what does your dog weigh” but due to incorrect translation, it states “breed category” in the English version. Even with this in mind we have reviewed the relevance of the matter and have chosen not to focus on this association as suggested. The wording in the translated questionnaire and in the revision has been corrected to “dog size in kg” instead of the previous “breed size”. The paragraph has been deleted.

Regarding "formulation type", in addition to the problem of potential confounding by dosage, this method of grouping is likely so heterogeneous that it may not be meaningful to cluster them together for analyses. For instance, the content and ratios of major cannabinoids (THC, CBD) by weight are equally important to consider. Best to side-step these issues by not concluding anything about whether formulation type or breed size affect owner-perceived efficacy. With a hypothesis-generating survey like this, it seems to me best to keep it simple and emphasize directions for future work rather than drawing highly questionable conclusions about your survey population.

Thank you for pointing this out. The paragraphs on formulation type and effect have been deleted.

This concludes my discussion of the broad issues I have with the paper. What follows are more specific issues (a number of which are fairly minor) that need to be addressed.

At times, the writing, grammar and spelling impeded my understanding. It would be useful to have a native English speaker read the manuscript for clarity, spelling, flow, and disambiguation. For example, "millenials" in line 51, "underlying incentive" line 88, "CDB" line 390, and the use of "over-all" and "further-more" throughout.

Thank you for the grammatical and spelling edit. The manuscript has been revised to the best of our ability.

Line 25 and elsewhere: there are numerous references to personal "experience", but no context for this (sounds like you are practicing medicine and clients are mentioning this to you). I think these statements detract from your overall rationale for performing the survey by suggesting that it may have been primarily motivated by the anecdotal reports of a handful of practicing veterinarians (which seems to be an unnecessarily weak rationale).

We have deleted all references to our personal experience. Thank you for the recommendation.

Lines 37-39: I would hesitate to generalize about dog owners in Denmark in general, since there is

likely a selection bias (as you have already highlighted). It would be safer to state that a large proportion of survey respondents supplement their dogs with cannabinoids.

We have amended the sentence.

Lines 82-89: I think you can strengthen your rationale for the study, by emphasizing that we aren't aware of all the potential indications that merit future study, and an anonymous survey is the best way to access this information, given the current legal situation.

Thank you. The rationale has been adjusted and the rationale has been amended to read; "By surveying the indications for the cannabinoid use, information will be generated on potential indications that merit for future research. Due to the legal status of using cannabinoids, an anonymous survey was found most suitable to obtain this information."

I don't think Fig 1 is necessary if this information is included in text, and for the reasons listed above about why formulation type isn't that meaningful. Moreover, almost all responses were in one category.

The figure has been removed.

For Figure 2, I don't think you need to provide a pie chart with the responses regrouped by "observed effect" vs. "no observed effect". I would argue that your original pie chart with the 4 types of responses does not require further simplification.

The figure has been revised.

Line 129, and elsewhere: I prefer "association" rather than "correlation". Although both are technically correct, "correlation" is most often used to describe associations between two linear variables (whereas you only have categorical variables).

Thank you for this point. Correlation has been substituted with association.

Lines 130-131: The "treatment indication" represents a nominal categorical variable, not an ordinal variable. Goodman-Kruskal's gamma is used to assess the association between two ordinal variables. I believe another statistic is needed in this case (Freeman's theta or epsilon-squared).

Thank you for this comment. We have consulted our statisticians and revised the statistical analysis for the association between effect and treatment indication to a Kruskal-Wallis test with epsilon-squared and Dunn's post hoc.

Lines 145-146: It's unclear how you performed your chi-square test. In the methods you stated that this would be used to test all geographic regions, yet in your results you only have 1 degree of freedom, implying only two groups were tested. Did you group all regions outside the capital region together and compare with the capital region? A chi-square test can't tell you whether one region is higher than another---how did you determine this? Logistic regression would be more appropriate to use in this case, given your interest in assessing which region(s) were most likely to have owners administering cannabinoids to their pets.

Thank you. In the original manuscript, pair-wise comparisons were performed between individual regions and a summarized group. We have gone back to our statisticians and

revised the statistical analysis to include a chi-square for test of the global effect and binominal logistic regression analysis for the multiple comparisons test.

Lines 145-146: can you speculate in the discussion about why respondents in the capital region were less likely to use cannabinoids?

We have included a paragraph in the discussion suggesting a possible explanation for this finding. The paragraph reads; “The study found that respondents living in Greater Copenhagen (Capital area) were less likely to supplement their dogs with cannabinoids. The reason for this is not clear, but it could be speculated to be linked to the higher educational level of the general population in this area, making these owners more adherent to evidence-based therapies, and less motivated for “alternative treatments”.

Lines 156-157: might be worth examining what indication these respondents used THC for? Anecdotal reports suggest it is useful for cancer.

Please see original paragraph line 262-264. This particular part has been removed from the revised manuscript as a consequence of the overall revision.

There are several instances where methods appear in the results section of the manuscript. For example, lines 173-177. The results should only be reporting what was found, not how the study was performed. Please correct.

Thank you for the structural comment. It has been corrected.

Tables 1 and 2 are redundant. Lines 187-190 are actually methods, and you can remove Table 1, and present only Table 2.

Thank you. Table 1 has been removed and lines 187-190 have been moved to the method section.

Even if you only perform statistics on the most numerous groups for indication vs. perceived efficacy (Table 3), I would include all groups in the table because it's still relevant and interesting for readers to see the perceived efficacy for other indications. You can remove Table 3 and bring Table S2 into the main manuscript to accomplish this.

Thank you for this suggestion. It has been changed.

Lines 282-283: this is quite a large number (167), how many of these respondents purchased through a veterinarian? This would be an interesting avenue to investigate. For example, which regions were these respondents in? For what conditions?

Thank you for your reflection. We have added these suggestions to the manuscript. The paragraph reads; Of the 20 respondents purchasing cannabinoids through veterinarians, the majority (n=15) was located in the Capital area (Greater Copenhagen, Northern Zealand, and Other Zealand). One or more indications for use were reported with the distribution: pain (n=12), behavioral issue (n=6), cancer (n=4), appetite (n=1), kidney disease (n=1), and allergy (n=1).

Lines 312: I'm not 100% certain about the situation in the US, but with the legalization of hemp,

there are supplement-type products that are now legally available for companion animals (CBD dog treats), but this might be restricted to certain states. Canada currently has no available licenced cannabinoid products for animals, veterinarians cannot legally prescribe human medicinal products off-label, and CBD-containing supplements for animals are not yet legal.

Lines 389-390: can you either clarify these terms or provide a reference where the readers can go to disambiguate these terms?

As a result of the general revision, this paragraph has been removed.

Line 390-391: based on this, it would be useful to examine the perceived efficacy for those respondents that used hemp seed oil. If this type of product doesn't contain cannabinoids, you wouldn't expect them to see a positive treatment effect. Consider removing these observations, since you are interested in cannabinoids, and not all products derived from cannabis plants.

We have chosen not to remove these responses or further examine them for effect trends. The reason for this is that the free-text often is too undetailed for us to be sure that “hemp seed oil” is an un-infused oil or if the respondent has neglected to type in “CBD infused hemp seed oil”.

Response to Reviewer #2

I was delighted to be asked to review this paper, it is an interesting and novel study which is highly valuable in the current rapidly changing field of cannabis in our pets. There are a fair few changes which need to be made to this manuscript before I can recommend it for publication.

Thank you for the very insightful comments. To the best of our ability we have revised the manuscript.

I didn’t see the raw data available as requested by the journal.

We do apologize for this error. The original and translated data have been included in the revision.

I think that the manuscript would benefit from being streamlined to make it easier to read and to follow.
I think that the manuscript would also benefit from being proofread for clarity and flow as it currently jumps around a bit which makes it hard to follow
I have pointed out some of the places where commas are needed but there are many, so reading the manuscript aloud will aid the readability of the paper

Thank you for the structural, grammatical, and punctuation comments. They have been corrected and the revised manuscript has been proof read.

Line 25- I think references to personal experience in the abstract is not the best. Would be nice not to see them so much throughout and perhaps reference or explain these more

Thank you for commenting on that. It has been removed from the manuscript.

Line 37- I think generalising for all dog owners in Denmark is a bit of a stretch based on the bias in the study.

Thank you for pointing this out. It has been rephrased; "This study shows that, despite that no licensed veterinary cannabinoid products are available in Denmark, dog owners do supplement their dogs with cannabinoids and that the majority of these perceive that the treatment had a positive effect."

Line 43- In recent years, there has … (add in comma)

Thank you for the grammatical edits.

Line 44-45- this reads a bit strange- please consider rewording

Thank you, it has been reworded; "Also among layman, there has been an interest in self-prescribing ''unlicensed" cannabinoid products for various medical conditions, these also including self-prescription to their companion animals."

Line 50- what is the legal status of cannabis in Denmark?

The paragraph has been revised in order to highlight this issue; "In Denmark recreational cannabis is prohibited, and up until 2018, medical cannabis products have not been available. In 2018 a national four-year pilot programme was passed by the Danish government, allowing physicians to prescribe medical cannabis for certain indications. The purpose of the programme was to enable prescription of medical cannabis to patients, who had no effect from conventional therapy. Veterinary use is not included in the pilot programme, and no registered medical cannabis products are available for animal use in Denmark."

Line 51- millennia rather than millennials?

Absolutely, thank you. It has been revised.

Line 75- comma after patient

Thank you for the grammatical edits.

Line 83- comma after we.

The section was deleted as part of the general revision.

I found the aims and hypothesis of the study a bit long winded and could be written a bit more clearly.

Thank you for pointing this out. The section has been revised and reads; "The aim of this study was to explore the use of cannabinoids in Danish dogs, by evaluating to what extent unauthorized cannabinoids are being used, for which indications they are used and the owners perceived effect."

Line 94-95- not sure that this is needed

Thank you for the comment, it has been revised and reads; “The survey was designed and reviewed by the co-investigators, and an online pilot questionnaire was tested by staff at the University of Copenhagen (UCPH).”

Line 101- comma after survey
I do not have concerns about the ethics here, but it is amazing how ethics differs between universities. It is a part of a wider issue which I feel needs to be addressed and streamlined to make it similar, as some universities will require full ethical approval for a study like this

Thank you for the very relevant observation, especially on the thoughts of streamlining ethical approval consensus. At the University of Copenhagen, a Data Protection Officer advises employees on the rules of data protection in regards to scientific data collection. The Officer did not find the survey including any personal data or sensitive data.

At the University of Copenhagen, a local Care and Use Committee (The Local Ethical and Administrative Committee) reviews and approves all studies performed at the Department of Veterinary Sciences. They have on application, prior to the study was performed, concluded that we did not need ethical approval, due to the nature of the data and respondent anonymity, and categorized the study as exempt. After your relevant comments, we have gone back to the Local Ethical and Administrative Committee and asked them to comment on this. They have revised their conclusion and have approved the study (study identification number: 2020-20).

Line 111- comma after positively

Thank you for the grammatical edits.

Line 129- is this a correlation? Do you have stats to back it up?

The statistics have been revised.

Line 143- comma after questionnaires

Thank you for the grammatical edits.

Line 145- I think having much more detail in the chi square will help

The statistics have been revised.

Line 145- is cannabis use in humans more common in the capital than in the other areas?

We do not have national statistics to back this up, but in the Capital region we do have a free-state where the purchase of recreational cannabis is easily assessable. National statistics show that the purchase of cannabis is highest in the Capital, but this probably does not reflect the use among inhabitants in the Capital.

Line 150- comma after used

The section has been removed due to general revision.

I am not entirely sure that figure 1 adds much to the manuscript

Thank you. After this revision, the figure has been removed.

Line 168- could you please add in what you mean by hash? I presume cannabis but please add in as this is not a word commonly used here.

Thank you for addressing this. Hash (hashish) is the resin collected from the cannabis flower and the word used by laymen for recreational marijuana when smoked in Denmark. We have changed the wording to marijuana, which, as you point out, is more appropriate in English.

Table 1- was any behavioural issues thought of for part of this study?

No, at the time of the study we did not have a focus on behavioral treatment. From the free text answers, we discovered a large proportion of respondents using it for that indication.

Line 200- we are aware that people take this drug sometimes to relax or because they want to. Is there any evidence that humans do that with their dogs too?

Not that we are aware of, except for the relaxational effect on stressed dogs. An often reported indication was stress or anxiety, so you could say that the owners gave the cannabinoids for relaxation, but we reckon that you are referring to recreational purposes, which we have not seen described.

There is quite a bit of methodology within the results which need removing

Thank you for pointing this out. We have to the best of our ability removed it and replaced it appropriately.

I am not sure that tables 1 and 2 add much. Table 2 adds more than table 1

Table 1 has been removed, but we have chosen to keep table 2.

I am also not convinced that figure 2 adds much to the manuscript

Thank you for your input, the figure has been revised and we have found it complementary for the text, so it is still included.

I would urge you to revisit the stats, I think that you can add more in which will strengthen the manuscript e.g. in table 3

Thank you for pointing this out. The statistics have been revised and table 3 substituted.

Line 283- if cannabis products are not licenced for animals why are vets selling them? And was there a reason why these animals were prescribed it?

An interesting point, that we are not able to answer. There are no licensed products for veterinary use in Denmark. We do speculate that some of the respondents are confusing hemp seed products for cannabinoid-containing products, but from their free texts it has not been possible to confirm this. Additional possibilities are that there are veterinarians selling unlicensed products. We have added further information for this subgroup on location and for what indications they use the veterinarian “prescribed” products.

I know it wasn't asked as a question, but it would be of interest to find out if there was a correlation between humans taking it and dogs taking it.

Thank you for the suggestion. We did choose not to include personal cannabis use in this questionnaire, due to GDPR concerns.

Line 304- this sentence reads awkwardly so please consider rewording

Thank you for the comment. The sentence has been revised.

Line 310- this sounds very high, 78% of all US dog owners use cannabis? It needs rewording or qualifying I think

Thank you for pointing this out. We have reworded the sentence; "In these studies, a higher proportion of respondents indicated use of cannabinoids, with percentages of cannabinoid use being 79.8 %[23] and 78.3 %[21]."

Please report decimal figures properly, as in 0.002

Thank you, it has been changed.

Line 390- can you put in something to define what the differences between these products is please?

The paragraph has been revised and the sentence removed due to general revision.

Thank you once again for the possibility to re-submit.

With kind regards
Pernille Holst
